# Selenocysteine Insertion Sequence Binding Protein 2L Is Implicated as a Novel Post-Transcriptional Regulator of Selenoprotein Expression

**DOI:** 10.1371/journal.pone.0035581

**Published:** 2012-04-17

**Authors:** Jesse Donovan, Paul R. Copeland

**Affiliations:** Department of Microbiology, Molecular Genetics, and Immunology, University of Medicine and Dentistry of New Jersey-Robert Wood Johnson Medical School, Piscataway, New Jersey, United States of America; University of Kent, United Kingdom

## Abstract

The amino acid selenocysteine (Sec) is encoded by UGA codons. Recoding of UGA from stop to Sec requires a Sec insertion sequence (SECIS) element in the 3′ UTR of selenoprotein mRNAs. SECIS binding protein 2 (SBP2) binds the SECIS element and is essential for Sec incorporation into the nascent peptide. SBP2-like (SBP2L) is a paralogue of SBP2 in vertebrates and is the only SECIS binding protein in some invertebrates where it likely directs Sec incorporation. However, vertebrate SBP2L does not promote Sec incorporation in *in vitro* assays. Here we present a comparative analysis of SBP2 and SBP2L SECIS binding properties and demonstrate that its inability to promote Sec incorporation is not due to lower SECIS affinity but likely due to lack of a SECIS dependent domain association that is found in SBP2. Interestingly, however, we find that an invertebrate version of SBP2L is fully competent for Sec incorporation *in vitro*. Additionally, we present the first evidence that SBP2L interacts with selenoprotein mRNAs in mammalian cells, thereby implying a role in selenoprotein expression.

## Introduction

Selenoproteins are unique in that they contain the amino acid selenocysteine (Sec). Commonly referred to as the 21st amino acid, Sec is encoded by UGA codons which typically specify translation termination. In order to recode UGA from stop to Sec, selenoprotein mRNAs contain a Sec insertion sequence (SECIS) element in their 3′ UTRs which is recognized by SECIS binding protein 2 (SBP2). Although mechanistic detail is lacking, the concerted action of SECIS-bound SBP2 and the Sec specific translation elongation factor (eEFSec) is essential for recoding UGA from stop to Sec and delivering Sec-tRNA^Sec^ to the ribosomal A-site [1]–[3].

Eukaryotic SECIS elements are stable stem-loop structures that belong to the kink-turn family of RNAs [2], [4]. They are comprised of two helices separated by an internal loop of 4–18 nucleotides [5]. The base of helix 2 contains 4 non-Watson-Crick base pairs including the tandem G.A/A.G “quartet” that with a 5′ RU forms the conserved SECIS core motif (AUGA) and is essential Sec incorporation [4], [6]–[9]. SECIS elements also have an apical motif comprised of three unpaired adenosines or cytidines that are required for Sec incorporation [5], [10], [11]. Additionally, there are two forms of SECIS elements: Form 1 SECIS elements have a large apical loop while Form 2 SECIS elements have a smaller apical loop that is separated from helix 2 by a bulge and third helix. No functional differences have been described for either SECIS form, but a recent survey found that Form 2 is more prevalent [5].

SECIS elements direct Sec incorporation upon recognition of the core motif by SBP2. Structure-function analyses have divided SBP2 into an N-terminal domain that is dispensable for Sec incorporation, a central Sec incorporation domain (SID), and a C-terminal RNA binding domain (RBD) that contains an L7Ae RNA binding motif [12]. The C-terminal half of SBP2 (CT-SBP2; rat a.a. 399–846, human a.a. 408–854) is sufficient to promote Sec incorporation. Both the SID and RBD are required for Sec incorporation activity, but a non-conserved region between the SID and RBD is dispensable as are residues downstream of the RBD (C-terminal of rat a.a. 777; human a.a. 783) [12], [13]. Additionally, residues in the C-terminus of the SID have been implicated in differential SECIS affinity and mediate stable SECIS-dependent interaction of the SID and RBD that appears necessary for Sec incorporation [13]–[15].

A homologue of SBP2, termed SBP2-like (SBP2L), was identified in BLAST searches when SBP2 was first cloned [16], and a subsequent survey of eukaryotic SECIS binding proteins (SBPs) found that SBP2 and SBP2L are paralogues in vertebrates while SBP2L is the sole SBP in some invertebrates including sea urchins, sea squirts, and an annelid worm in the genus *Capitella* [17]. Since invertebrate SBP2L is the only SBP in these organisms it likely fulfills the known functions of mammalian SBP2. Although selenoproteins have not been detected by metabolic labeling with ^75^Se in these organisms, they have been detected by *in silico* sequence analyses [17]–[19]. Additionally, a deiodinase cDNA including the SECIS was cloned from a sea squirt, *Halocynthia roretzi*, and its enzymatic activity in transfected mammalian cells depended on the Sec codon [20], providing indirect support for invertebrate SBP2L as a Sec incorporation factor. This notion stands in contrast to the observation that a C-terminal fragment of human SBP2L (CT-SBP2L; a.a. 467–1101) that is homologous to CT-SBP2 cannot direct Sec incorporation *in vitro* [12], [17]. Although SBP2 and SBP2L are similar, there are several defining features that clearly distinguish the proteins. These include five sequence motifs surrounding the SID and RBD as well as a poly-glutamate motif C-terminal to the RBD that are exclusively found in SBP2L [17]. Thus, with the exception of a short N-terminal motif, conservation between SBP2 and SBP2L is limited to the SID and RBD [17], [21]. The conservation between vertebrate SBP2L, invertebrate SBP2L, and mammalian SBP2, point toward a role for SBP2L in the post-transcriptional regulation of selenoprotein expression and it has been postulated that SBP2L may direct selenocysteine incorporation for a subset of the vertebrate selenoproteome [17].

In this report we used *in vitro* RNA binding assays to determine the affinities of human SBP2 and SBP2L for the 26 known human SECIS elements. Additionally, we found that the SID and RBD of SBP2L, unlike those of SBP2, do not interact in a SECIS dependent manner thereby explaining its inability to promote Sec incorporation *in vitro*. Interestingly, SBP2L from the worm *Capitella teleta* is able to promote robust Sec incorporation, confirming the role of SBP2L as the Sec incorporation factor in this organism. Lastly, we found that selenoprotein mRNAs co-immunoprecipitate with both SBP2 and SBP2L suggesting the existence of at least two selenoprotein mRNP populations and implicating SBP2L in the post-transcriptional regulation of selenoprotein expression.

## Results

### *Capitella* SBP2L promotes Sec incorporation

**Figure 1:**
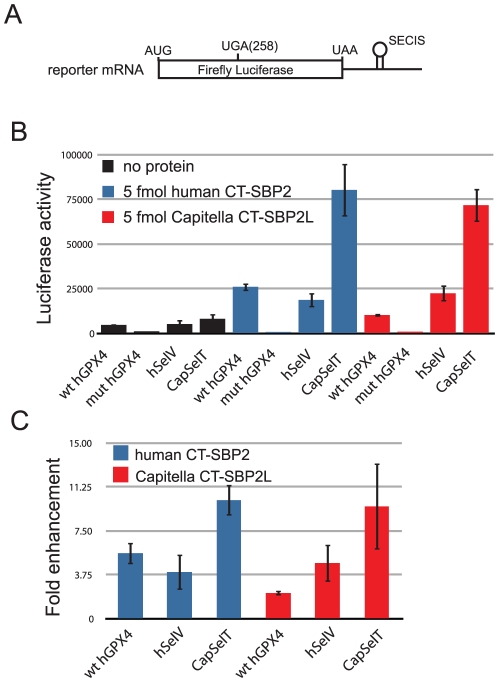
*Capitella* CT-SBP2L is able to promote Sec incorporation. (A) Diagram of the luciferase Sec incorporation reporter construct. (B) 5 fmol of *in vitro* translated CT-SBP2 (blue bars) or *Capitella* CT-SBP2L (red bars) were added to a rabbit reticulocyte lysate assay programmed with a luciferase reporter construct containing a Sec codon at position 258 and the SECIS element indicated. The data is reported as the mean ± standard deviation of three experiments (except for the mutant GPX4 SECIS element, which was only tested once). (C) The data from (B) was replotted to show the fold enhancement of Sec incorporation upon the addition of either CT-SBP2 (blue bars) or *Capitella* CT-SBP2L (red bars).

We have previously demonstrated that mammalian SBP2L lacks Sec incorporation activity in a rabbit reticulocyte lysate *in vitro* translation assay [12], [17]. However, since some invertebrate organisms possess only SBP2L, we decided to test the ability of one such SBP2L to promote Sec incorporation in an *in vitro* translation assay. Based on genomic and expressed sequence tagged sequences, we synthesized the portion of *Capitella* SBP2L that corresponds to the active C-terminal fragment of mammalian SBP2 (Figure S1). This protein was *in vitro* translated in rabbit reticulocyte lysate, quantitated, and 5 fmol was added to a separate *in vitro* translation reaction programmed with a luciferase reporter mRNA that harbored various SECIS elements: wild-type or mutant human glutathione peroxidase 4 (GPX4, a Form 1 SECIS element), human selenoprotein V (SelV, a Form 2 SECIS element) and *Capitella* selenoprotein T (SelT, Form 2; diagrammed in Figure 1A). The same experiment was performed with human CT-SBP2 as a positive control. As shown in Figure 1B, *Capitella* CT-SBP2L is able to support Sec incorporation from all three SECIS elements to varying extents, but no Sec incorporation was detected from a GPX4 SECIS element that harbored an AUGA→AUCC mutation. It is interesting to note that the *Capitella* SelT SECIS element, which has not previously been tested, appears to permit Sec incorporation with approximately a three-fold higher efficiency, but both CT-SBP2 and *Capitella* CT-SBP2L enhance *Capitella* SelT based Sec incorporation to the same extent (Figure 1C). It is also notable that *Capitella* CT-SBP2L shows an impaired ability to stimulate GPX4-based Sec incorporation. Interestingly, both the human SelV and *Capitella* SelT SECIS elements are of the form 2 variety, while human GPX4 is form 1. In addition, since no form 1 SECIS elements have been identified in invertebrates, it is possible that *Capitella* CT-SBP2L is inherently less active with form 1 SECIS elements. Overall these results clearly establish that *Capitella* SBP2L is able to promote Sec incorporation in a SECIS-core dependent fashion to approximately the same extent as human CT-SBP2. While these results do not directly shed light on the function of mammalian SBP2L, it is clear that possessing the conserved SBP2L features is not sufficient to prevent Sec incorporation activity.

### Recombinant CT-SBP2L does not promote Sec incorporation

**Figure 2:**
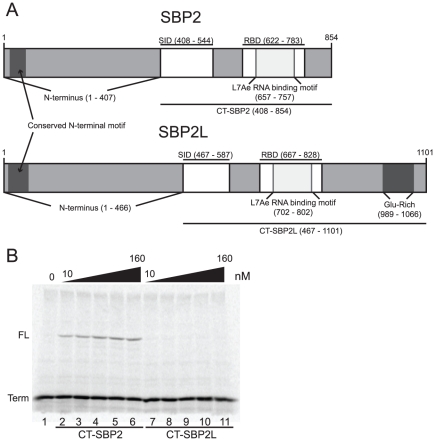
CT-SBP2L does not promote Sec incorporation. (A) Domain architecture of human SBP2 and SBP2L. The conserved Sec incorporation domains (SID) and RNA binding domains (RBD) are indicated. The C-terminal (CT−) portions of the proteins used through this study are underlined. (B) A representative gel showing [^35^S]-Met labeled translation products of the Sec incorporation reporter mRNA in the presence of the indicated recombinant proteins. Full-length (FL) product is the result of Sec incorporation while the termination product (Term) results from translation termination at the in-frame UGA/Sec codon.

Previous studies showed that limiting amounts of *in vitro* translated human CT-SBP2L (Figure 2A; a.a. 467–1101) could not promote Sec incorporation when rabbit reticulocyte lysate was programmed with mRNA encoding the selenoprotein GPX4 or a luciferase reporter mRNA containing an in-frame UGA codon and rat GPX4 SECIS element in the 3′ UTR [12], [17]. In those cases, the amount of SBP2L was limited by *in vitro* translation, so in order to investigate a full range of CT-SBP2L concentrations, we tested the ability of recombinant CT-SBP2L to promote Sec incorporation in a luciferase reporter mRNA. The reporter mRNA was translated in rabbit reticulocyte lysate in the presence of [^35^S]-Met and 0–160 nM recombinant human CT-SBP2 or CT-SBP2L and aliquots of the translation reactions were analyzed by SDS-PAGE and phosphorimaging. As expected, only the translation product resulting from termination at the Sec codon was visible in the absence of added protein (Figure 2B, lane 1; Term). The addition of recombinant human CT-SBP2 resulted in the production of full-length luciferase due to Sec incorporation at the UGA codon (lanes 2–6; FL). Consistent with previous results, CT-SBP2L did not promote Sec incorporation at any of the concentrations tested (lanes 7–11). In addition, it is noteworthy that increasing concentrations of CT-SBP2L did not alter the amount of termination product suggesting that SBP2L neither destabilizes selenoprotein mRNAs nor inhibits translation initiation (compare termination product in lane 1 to lanes 7–11). These results further support the idea that mammalian SBP2L does not directly participate in the Sec incorporation reaction in a manner analogous to that of SBP2.

### SBP2L has lower affinity for SECIS elements than SBP2

**Figure 3:**
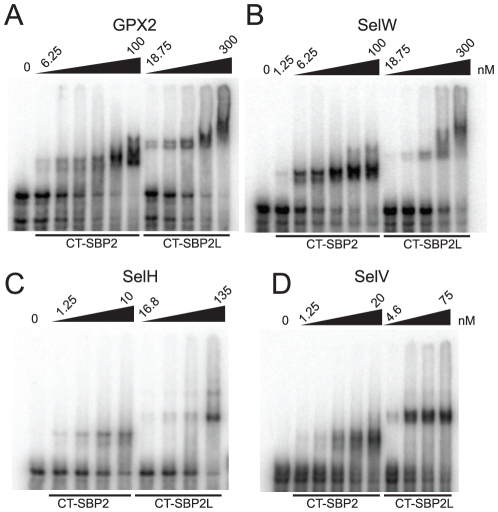
SBP2L is a universal SECIS binding protein. Representative EMSA gels of CT-SBP2 and CT-SBP2L with the human GPX2 (A), SelW (B), SelH (C), and SelV (D) SECIS elements.

**Figure 4:**
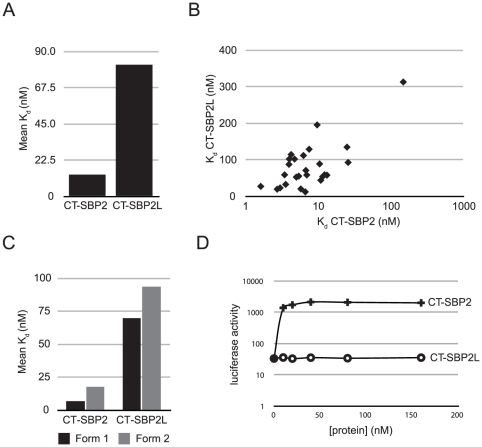
Summary of SECIS binding data. (A) The mean apparent dissociation constants (K*_d_*) for CT-SBP2 and CT-SBP2L obtained from the values reported in Table 1. (B) Scatterplot of CT-SBP2L SECIS dissociation constants as a function of CT-SBP2 SECIS dissociation constants. (C) Mean dissociation constants of CT-SBP2 and CT-SBP2L for Form 1 and Form 2 SECIS elements. (D) Total luciferase activity from a luciferase Sec incorporation reporter construct containing the human SelV SECIS element. A range of recombinant CT-SBP2 or CT-SBP2L (10–160 nM) was added as indicated.

We have previously shown that SBP2L is a bona fide sequence-specific SECIS binding protein albeit with a lower apparent binding affinity for the rat GPX4 SECIS element [12]. A subsequent analysis of SBP2/SBP2L chimeric proteins led us to hypothesize that vertebrate SBP2L may promote Sec incorporation for a subset of the selenoproteome [17]. Thus, we set out to determine the affinities of human SBP2 and SBP2L for all known human SECIS elements with two questions in mind: 1) Does SBP2L have a higher affinity than SBP2 for any SECIS elements and 2) If so, can SBP2L promote Sec incorporation using a SECIS element for which it has higher affinity than SBP2? We used an electrophoretic mobility shift assay (EMSA) to determine the apparent dissociation constant (K*_d_*) of each human SECIS element for recombinant CT-SBP2 and CT-SBP2L. The concentration of recombinant protein required to shift 50% of the labelled SECIS RNA was considered to be the apparent K*_d_*. Several representative EMSAs used for determining K*_d_* values are shown in Figure 3. The dissociation constants of all the SECIS elements with CT-SBP2 and CT-SBP2L are listed in Table 1. We found that CT-SBP2L and CT-SBP2 bound all SECIS elements. CT-SBP2L has the highest affinity for the SelV SECIS (K*_d_* = 12.4 nM) and the lowest affinity for the GPX6 SECIS (K*_d_* = 313.2 nM) while CT-SBP2 has the highest affinity for selenoprotein P SECIS 2 (K*_d_* = 1.6 nM) and the lowest affinity for the GPX6 SECIS (Kd = 149.8 nM). Overall CT-SBP2L has a lower affinity for SECIS elements with an average K*_d_* of 81.7 nM while that of CT-SBP2 is 13.5 nM (Figure 4A). Additionally CT-SBP2L was unable to bind any SECIS element with higher affinity than CT-SBP2. Visualizing the affinities of SECIS elements for CT-SBP2L as a function of affinity for CT-SBP2 showed that there is no relationship between SECIS element binding affinities for SBP2 and SBP2L (Figure 4B). Since SECIS elements exist in two major classes, Form 1 with a single terminal loop and Form 2 with a minihelix separating a bulge from the apical loop, we also examined whether or not there was a relationship between SECIS form and SBP affinity. The average K*_d_* values of CT-SBP2 and CT-SBP2L for Form 1 SECIS elements were lower than that for all SECIS elements (6.8 nM and 69.7 nM, respectively) while that for Form 2 SECIS elements were higher than the overall average (17.6 and 93.6 nM, respectively; Figure 4C). We found no relationship between CT-SBP2 and CT-SBP2L affinities for Form 1 or Form 2 SECIS elements (data not shown). These results demonstrate that both SBP2 and SBP2L bind all known SECIS elements with a wide range of affinities but do not yield any distinction between Form 1 and 2 SECIS elements. The overlapping ranges of SECIS binding affinities for CT-SBP2 and CT-SBP2L suggest that the inability of CT-SBP2L to promote Sec incorporation is not due to poor SECIS binding. This was experimentally verified by testing the ability of CT-SBP2L to promote Sec incorporation with the human SelV SECIS element. To that end, we sub-cloned the human SelV SECIS element into the luciferase Sec incorporation reporter. Figure 4D shows that even across a broad range of CT-SBP2L protein (10–160 nM) concentrations, no Sec incorporation is detectable. The same range of CT-SBP2 showed saturating Sec incorporation activity at approximately 40 nM.

**Table 1:** Apparent dissociation constants of CT-SBP2 and CT-SBP2L for all human SECIS elements.

	Apparent K*_d_* (nM)*		
SECIS	CT-SBP2	CT-SBP2L	Form
GPX1	6.3±0.1	112.1±11	1
GPX2	10.9±2.3	135.0±26.1	1
GPX3	25.0±13.8	135.0±26.2	2
GPX4	13.2±2.8	58.2±2.9	1
GPX6	149.8±39.5	313.2±31.8	2
SEP15	5.0±0.4	52.9±12.9	2
SelH	4.7±0.6	102.4±11.8	1
SelI	12.2±1.7	59.2±5.5	2
SelK	3.6±2.0	32.9±4.6	2
SelM	3.0±0.1	23.4±0.1	2
SelN	10.5±0.1	89.0±3.5	1
SelO	7.0±0.7	58.7±9.3	2
SelP SECIS 1	3.4±0.1	59.2±5.3	2
SelP SECIS 2	1.6±0.0	27.6±3.6	1
SelR	9.7±0.2	195.8±21.3	2
SelS	25.9±6.7	93.0±4.4	2
SelT	4.0±0.2	102.3±2.0	1
SelV	6.7±0.2	12.4±0.4	1
SelW	4.0±0.2	87.7±8.4	2
SPS2	7.5±0.6	129.2±19.8	1
DIO1	2.7±0.9	19.9±4.8	1
DIO2	6.8±3.5	71.3±33.7	2
DIO3	5.4±2.2	55.7±19.6	2
TR1	5.8±0.7	20.6±0.5	2
TR2	11.6±0.9	54.4±2.0	2
TR3	4.2±0.2	114.7±19.8	2

* Mean ± standard deviation; n = 2.

**Figure 5:**
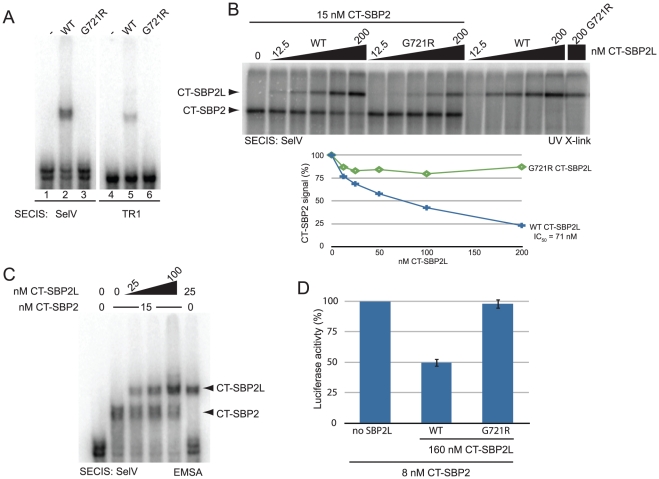
SBP2 and SBP2L compete for SECIS binding *in vitro*. (A) EMSA of 20 nM wild-type (WT) or mutant (G721R) CT-SBP2L with the indicated SECIS elements. (B) Top: UV Cross-linking (X-link) of CT-SBP2 and CT-SBP2L. Bottom: Quantitation of CT-SBP2 signal in the presence of increasing concentrations of CT-SBP2L. (C) EMSA of the SelV SECIS element with CT-SBP2 in the presence of increasing amounts of CT-SBP2L. (D) Sec incorporation activity in the presence of 8 nM CT-SBP2 plus 160 nM wild-type or G721R mutant CT-SBP2L. Luciferase activity from 1 nM luciferase mRNA harboring the SelV SECIS element is normalized to a reaction containing no CT-SBP2L (left bar).

In order to verify that the SBP2L-SECIS interaction is driven by the conserved L7Ae motif, we tested the SECIS binding activity of mutated CT-SBP2L. The L7Ae motif in the SBP2 RNA binding domain recognizes the SECIS core and mutating a universally conserved glycine within the L7Ae motif to arginine disrupts SBP2-SECIS interactions [12]. Since SBP2L-SECIS interactions are also SECIS core-dependent [12], [17], we reasoned that mutating the conserved glycine in the SBP2L L7Ae motif (G721) to arginine would inhibit SECIS binding. Indeed, the G721R mutation in CT-SBP2L inhibited binding to both the SelV and thioredoxin reductase 1 (TR1) SECIS elements (Figure 5A). This suggests that SBP2 and SBP2L have an overlapping, if not the same, binding site on the SECIS element. We further tested the impairment of SECIS binding activity in the G721R mutation in competitive SECIS binding assays using UV crosslinking. As shown in Figure 5B, the signal generated from UV cross-linking radiolabeled SECIS element to CT-SBP2 decreases with increasing concentrations of wild type but not mutant CT-SBP2L with a concentration of 71 nM at 50% inhibition (IC50). Note that the residual UV crosslinked signal observed in the G721R CT-SBP2L mutant is not unexpected, as this assay permits the observation of non-specific and transient RNA protein interactions. Interestingly, the same competitive assay when performed in an EMSA showed that SBP2 and SBP2L form separate complexes without a supershift indicating that they do not simultaneously interact with the same SECIS element (Figure 5C). It follows from these results that CT-SBP2L should inhibit Sec incorporation in vitro when present at concentrations high enough to compete for SBP2 binding to the SECIS element. Indeed, Figure 5D shows that wild-type but not the G721R mutant CT-SBP2L is able to reduce Sec incorporation with an IC50 of ∼160 nM. The fact that this IC50 is more than twice that observed in the UV crosslinking experiment (Figure 5B), suggests that the SBP2/SECIS interaction may be stabilized by other factors in the rabbit reticulocyte lysate. Together these data indicate that both SBP2L and SBP2 interact with a SECIS element by a similar mechanism, suggesting that SBP2L cannot support Sec incorporation because of a defect downstream of SECIS binding.

### SBP2L SID-RBD interactions differ from those in SBP2 and provide a mechanistic explanation for lack of Sec incorporation

**Figure 6:**
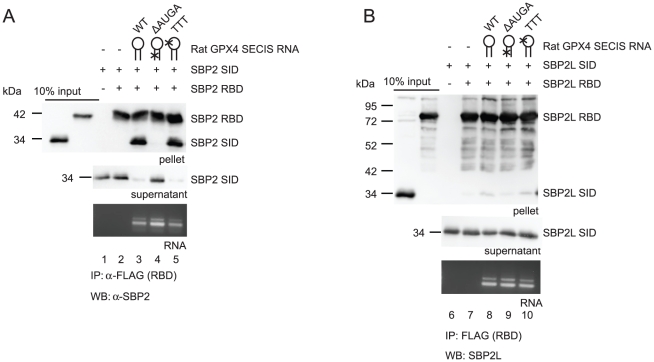
The SBP2L SID and RBD do not stably interact. (A) 6xHis-Xpress tagged rat SBP2 SID was incubated with FLAG tagged rat SBP2 RBD and wild-type (WT), core deleted (ΔAUGA), or loop mutant (TTT) rat GPX4 SECIS as indicated and complexes were immunoprecipitated (IP) with α-FLAG agarose. Top western blot: 50% pellet; middle western blot: 7.5% supernatant; bottom panel: RNA extracted from 50% of the supernatant was resolved on an agarose gel. (B) Same as in panel A but 6xHis-Xpress tagged SBP2L SID was incubated with 6xHis-FLAG tagged SBP2L RBD.

We previously reported that the SBP2 Sec incorporation and RNA binding domains (SID and RBD, respectively) interact in a SECIS dependent manner and are fully competent for Sec incorporation when expressed as separate proteins [14]. Since recombinant and *in vitro* translated SBP2L do not promote Sec incorporation, we set out to determine whether or not a lack of domain interaction between the SBP2L SID and RBD may explain the lack of Sec incorporation activity. To test this we subcloned SBP2L fragments that contain the SID (a.a. 467–647) and RBD (a.a. 648–1101) and expressed them as N-terminally His-Xpress and His-FLAG tagged recombinant proteins, respectively. SID-RBD interactions were assayed by immunoprecipitating the RBD with α-FLAG agarose in the presence or absence of wild-type or mutant rat GPX4 SECIS elements. We used N-terminally His-Xpress tagged rat SBP2 SID (a.a. 399–585) and FLAG tagged RBD (a.a. 586–846) as a positive control. As shown in Figures 6A and B neither the SBP2 nor SBP2L SID immunoprecipitates in the absence of RBD and SECIS (lanes 1 and 6). The SID from both proteins also does not pellet when incubated with RBD alone (Figures 6A and B; lanes 2 and 7). As expected, the SBP2 SID was pulled down by the SBP2 RBD in the presence of wild-type but not the ΔAUGA SECIS mutant (Figure 6A; lanes 3 and 4). The SBP2 SID also interacted with the SBP2 RBD in the presence of the apical loop mutant SECIS element (Figure 6A; lane 5). Surprisingly, the SBP2L SID showed dramatically reduced co-immunoprecipitation with the SBP2L RBD in the presence of wild-type or mutant SECIS elements (Figure 6B; lanes 8–10). Western blot analysis of the supernatant showed that the SBP2L SID was stable throughout the experiment eliminating protein degradation as a reason for lack of SID pelleting (Figure 6B, lanes 6–10, middle panel). The lack of SBP2L SID pelleting cannot be explained by RNA degradation as all SECIS RNAs were recovered from the supernatant after immunoprecipitation (Figure 6B, bottom panel). We repeated this assay with the human GPX4 and SelV SECIS elements and found that neither SECIS could promote stable association of the SBP2L SID and RBD (Figure S2).

These results suggest that SBP2L may not participate in Sec incorporation due to a lack of stable SID-RBD interactions and raise the question of whether or not it would be able to promote Sec incorporation if such a stable interaction could be induced. To this end we considered differences between CT-SBP2 and CT-SBP2L. The region between the SID and RBD is conserved in SBP2L but not in SBP2 and SBP2L has a conserved C-terminal extension relative to SBP2 that contains a Glu-rich motif [17]. Furthermore, deletion of the region between the SID and RBD did not affect the ability of SBP2 to participate in Sec incorporation [13]. We set out to determine if deletion of the inter-domain region (Δ602–663) or deletion of the C-terminal extension (Δ829–1101) could activate CT-SBP2L. Neither mutant, alone or together, conferred Sec incorporation activity to CT-SBP2L, suggesting that these regions do not contain inhibitory domains (Figure S3). These results are consistent with our finding that *Capitella* CT-SBP2L, which also contains these domains, is fully active for Sec incorporation *in vitro* and they reinforce the idea that mammalian SBP2L is not directly involved in Sec incorporation.

### Mammalian cells have two distinct selenoprotein mRNP populations

**Figure 7:**
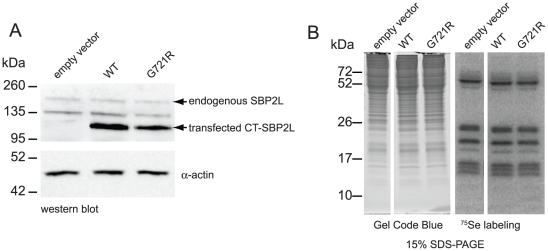
Overexpressing CT-SBP2L does not alter selenoprotein expression. HEK293 cells were transiently transfected with empty vector, wild-type (WT), or G721R CT-SBP2L expression vectors and labeled for 24 h with ^75^Se. (A) Equal amounts of cell lysates were analyzed by western blotting for SBP2L and β-actin. (B) Equal amounts of cell lysates were fractionated by SDS-PAGE. The gel was stained (left) to visualize total protein and ^75^Se labeled proteins were detected by phosporimaging (right).

**Figure 8:**
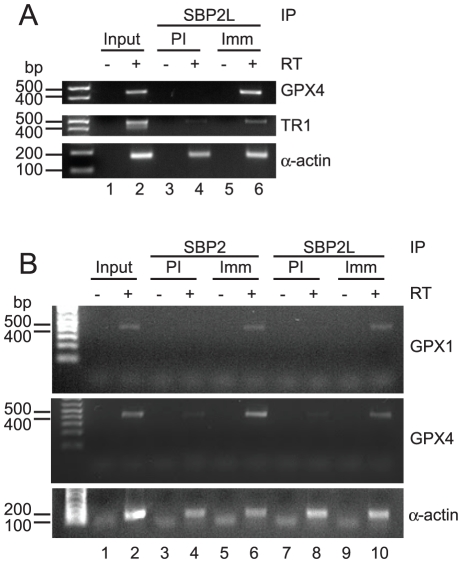
Association of selenoprotein mRNAs with SBP2L in mammalian cells. (A) Qualitative RT-PCR analysis of selenoprotein mRNAs (GPX4 and TR1) and β-actin mRNA extracted from pre-immune (PI) and immune (Imm) immunoprecipitations (IP) of SBP2L from U87MG cytoplasmic extracts. (B) Qualitative RT-PCR analysis of selenoprotein mRNAs (GPX1 and GPX4) and β-actin mRNA extracted from pre-immune (PI) and immune (Imm) immunoprecipitations (IP) of SBP2 and SBP2L from PC3 cytoplasmic extracts. RT, reverse transcriptase.

To date all attempts to determine SBP2L function have relied on *in vitro* assays which may not reproduce conditions required for proper function. Therefore we hypothesized that altering SBP2L expression in mammalian cells could affect selenoprotein expression. First, we transiently transfected HEK293 cells with vectors expressing wild-type or G721R CT-SBP2L followed by metabolic labeling of selenoproteins with ^75^Se. Over-expression of CT-SBP2L did not alter endogenous selenoprotein expression (Figure 7A and B), and we were unable to express detectable full-length SBP2L (data not shown). Additionally, we attempted SBP2L knockdown using shRNAs, but we achieved only modest reduction of SBP2L in HEK293 cells stably transfected with an shRNA vector targeting SBP2L. Metabolic labeling of these cells with ^75^Se showed that SBP2L knockdown did not affect selenoprotein expression (data not shown). Despite these data there remains a strong evolutionary argument that SBP2L is a factor that post-transcriptionally regulates selenoprotein expression. Therefore, selenoprotein mRNAs might be associated with SBP2L *in vivo*. We tested this hypothesis by co-immunoprecipitation from U87MG human glioblastoma cells as this cell line has higher expression levels of SBP2L compared to more commonly used cells lines (i.e. HeLa, HEK293) according to microarray data from the human gene atlas available through BioGPS [22], [23]. Prior to determining whether or not selenoprotein mRNAs co-IP with SBP2L we verified that our antibody can IP SBP2L (Figure S4). We cultured U87MG cells and immunoprecipitated SBP2L from cytoplasmic extracts. RNA was extracted from half of the pellet and the other half analyzed for protein content by mass spectrometry. Proteomic analysis detected SBP2L but not SBP2 (data not shown), verifying the lack of cross-reactivity between the anti-SBP2L antibody and human SBP2. This is further supported by lack of SBP2 immunoreactivity on western blots of SBP2L immunoprecipitations (data not shown). We performed RT-PCR on the RNA samples and found that mRNA of the selenoproteins GPX4 and TR1 were enriched in the α-SBP2L sample compared to the pre-immune sample while β-actin mRNA non-specifically immunoprecipitated with both the immune and pre-immune antibodies (Figure 8A; lanes 4 and 6). Additionally, we immunoprecipitated SBP2 or SBP2L from PC3 human prostate cancer cell cytoplasmic extract. RT-PCR analysis of these immunoprecipitations showed specific association of selenoprotein mRNAs (GPX1 and GPX4) with SBP2 (Figure 8B; compare lanes 4 and 6) and SBP2L (Figure 8B; compares lanes 8 and 10). These results implicate SBP2L in the post-transcriptional regulation of selenoprotein expression and demonstrate that mammalian cells have at least two selenoprotein mRNP populations.

## Discussion

In this report we investigated the potential role of SBP2L in regulating selenoprotein expression. We initially hypothesized that SBP2L might direct Sec incorporation for a subset of the selenoproteome and that this could be uncovered by analyzing the SECIS binding properties of SBP2L and comparing them to that of SBP2. Ultimately we were unable to demonstrate SBP2L participation in Sec incorporation but showed an association of selenoprotein mRNAs with SBP2L in cell extracts supporting a role for SBP2L in the post-transcriptional regulation of selenoprotein expression. In addition, the fact that the evolutionarily more “ancient” form of SBP2L from *Capitella* is fully active for Sec incorporation *in vitro* supports the conclusion that mammalian SBP2L is not directly involved in the Sec incorporation reaction, but the possibility that mammalian SBP2L plays a role under specific conditions cannot be ruled out.

### SECIS binding: SBP2 versus SBP2L

SBP2 and SBP2L comprise the SECIS binding protein family that possess the characteristic Sec incorporation domain and an RNA binding domain containing an L7Ae RNA binding motif. This study and previous works demonstrated that the interaction of SBP2 and SBP2L with SECIS elements is dependent on the SECIS core motif, suggesting that these proteins have the same binding site on the SECIS element. This is supported by several observations: 1) mutation of a universally conserved glycine within the L7Ae motif disrupts SECIS binding by SBP2 and SBP2L (this study and [6], [12]), 2) SBP2 residues predicted to contact SECIS RNA [6] are conserved between SBP2 and SBP2L [17], and 3) SBP2L competes with SBP2 for SECIS binding *in vitro* (Figure 4). Extending this competitive binding to the intracellular milieu suggests that there is one SBP per SECIS element at a given time and silver stained SDS-PAGE gels of eluates from large-scale immunoprecipitations suggest that SBP2 and SBP2L are not part of the same complex (Donovan and Copeland, unpublished observation).

Although they share the same binding site, the differing affinities for SECIS elements displayed by SBP2 and SBP2L suggest their binding mechanisms may be different. An IILKE motif and nearby residues in the C-terminus of the SBP2 SID are known modulators of SECIS affinity [13]–[15]. These residues are well conserved between SBP2 and SBP2L suggesting a minimal role in explaining the observed differences between SBP2 and SBP2L SECIS affinities. Gagnon et al. recently analyzed signature residues in L7Ae and its eukaryotic homologue, 15.5 kDa protein, for their contributions to K-turn and K-loop RNA binding [24]. Archaeal L7Ae can recognize K-turn and K-loop motifs in archaeal box C/D and box H/ACA while 15.5 kDa protein is limited to binding K-turn motifs in small nucleolar RNAs. The authors identified a single residue upstream of the conserved glycine and a downstream tetrapeptide motif that are determinants of RNA ligand recognition [24]. The equivalent residues are R672 and KAVP^740–743^ in human SBP2 and R717 and KLVP^785–788^ in human SBP2L. Structure modeling using the human 15.5 kDa protein bound to its target U4 RNA as a model (PDB coordinates 1E7K), suggests these signature motifs in SBP2 and SBP2L would be positioned near the 5′ side of the SECIS core and the variably sized internal loop of the SECIS element (Donovan and Copeland, unpublished observation). Thus it is tempting to speculate that the bulkier leucine residue in the SBP2L L7Ae RNA binding motif could contribute to the observed differences in SBP2 and SBP2L SECIS binding affinities.

### A connection between SECIS affinity and function?

We initially hypothesized that SBP2L might promote Sec incorporation in the presence of SECIS elements for which it has high affinity. However, Latrèche et al. recently tested the UGA recoding ability of all human SECIS elements and reported that there was no relationship between SBP2-SECIS affinity and recoding efficiency based on dissociation constants of SBP2 for the SelR and GPX3 SECIS elements which were used as representative strong and weak SECIS elements, respectively [25]. We obtained similar dissociation constants for SBP2-SelR and SBP2-GPX3 complexes and comparison of all SECIS-SBP2 affinities with the recoding efficiencies reported by Latrèche and colleagues reiterates the lack of correlation between SBP2 affinity and UGA recoding efficiency: DIO2, SelV, and SelO were classed as strong, moderate, and weak SECIS elements, respectively [25], but have similar affinities for SBP2 (Table 1). Similarly, there is no apparent relationship to SBP2L-SECIS affinity and recoding efficiency: SelP SECIS 1 and SelO are strong and weak SECIS elements, respectively, but have approximately the same affinity for SBP2L (∼60 nM) while another strong SECIS element, SelR, has the second lowest affinity for SBP2L (195 nM). Together these observations suggest that competitive SECIS binding based solely on affinity derived from in vitro experiments does not affect selenoprotein expression. This is also supported by CT-SBP2L overexpression data (discussed below).

### SBP2L as a post-transcriptional regulator

We attempted to address the function of SBP2L in human cell lines by manipulating expression levels of SBP2L in HEK293 cells. Our attempt to over-express full length SBP2L by transient transfection was unsuccessful and was not rectified by cloning an intron back into the SBP2L cDNA despite having similar transfection efficiency as CT-SBP2L (data not shown). Transient over-expression of CT-SBP2L did not affect selenoprotein expression suggesting that SBP2L does not inhibit selenoprotein expression by competing for SECIS elements with SBP2. This phenomenon has been observed for an unrelated RNA binding protein, eIF4A3, that selectively inhibits GPX1 expression by competing with SBP2 for SECIS binding [26]. Since over-expression of CT-SBP2L did not reduce selenoprotein expression, it also suggests that it does not increase turnover rates of selenoprotein mRNAs. SBP2L knockdown also did not affect selenoprotein expression. However, this lack of an effect could be explained by insufficient knockdown or potentially redundant functions in SBP2 masking any effect.

We also demonstrated for the first time that selenoprotein mRNAs co-immunoprecipitate with SBP2L from mammalian cytoplasmic extracts thereby implicating it as a post-transcriptional regulator of selenoprotein expression and establishing two distinct selenoprotein mRNPs that can be defined by their respective SECIS binding protein. The relatively slower evolutionary rate of vertebrate SBP2L relative to SBP2 and its conservation with invertebrate SBP2L [17], suggests that it too may function as a Sec incorporation factor, despite the lack of biochemical evidence. It has been reported, however, that a chimeric protein comprised of the SBP2 SID and the SBP2L RBD had some Sec incorporation activity, suggesting that the SBP2L RBD is capable of participating in Sec incorporation [17]. Based on our knowledge of SBP2 it is reasonable to predict that SBP2L function requires a stable SECIS dependent SID-RBD interaction. Indeed, despite our conclusion that SBP2L does not participate directly in Sec incorporation, it is possible that it does so in a highly regulated fashion. For example, it may be that in order for SBP2L-dependent Sec incorporation to occur, the protein undergoes activation, possibly phosphorylation by a signaling cascade, that would permit the SID and RBD to stably interact. Querying the Phosphosite database [27] revealed that phospho-SBP2L has been detected by mass spectrometry in several high throughput studies and one modification, phospho-Y818, is immediately downstream of the L7Ae RNA binding motif and could be involved in regulating activity. Additionally, microarray data from the human gene atlas [22], available at BioGPS [23], suggests that SBP2L expression is enriched in the central nervous system (CNS) and thyroid while SBP2 expression is enriched in testis, thyroid, and immune cells. This difference in tissue distribution could point toward SBP2L as a specialized Sec incorporation factor in the CNS while SBP2 acts a generalized Sec incorporation factor.

### Conclusions

Our results highlight fundamental differences between SBP2 and SBP2L, particularly the lack of stable SBP2L SID-RBD interactions. Additionally, the differential SECIS binding of SBP2 and SBP2L could set the stage for using these proteins and RNA ligands as new models for understanding how RNA binding proteins recognize their targets. Implicating SBP2L as a post-transcriptional regulator of selenoprotein expression is a critical first step toward deciphering SBP2L function.

## Materials and Methods

### Constructs

The CT-SBP2L clone in pCR3.1 used for producing *in vitro* translated protein has been described previously [12] as served as a template for subcloning the CT-SBP2L coding into pTrcHis and for generating the CT-SBP2L deletion mutants by site-directed mutagenesis. The CT-SBP2L coding region was PCR amplified without its stop codon and TOPO-TA cloned into pcDNA3.1 (Invitrogen) to generate C-terminally 6xHis-V5 tagged constructs for transfection. A full-length human SBP2L clone (BC033001) was obtained from ATCC and the coding region was TOPO-TA cloned into pcDNA3.1 (Invitrogen). Sequence analysis revealed that the original ATCC clone had a mutation that generated an L756F substitution which was corrected by site-directed mutagenesis. The G721R SBP2L mutant was obtained by site-directed mutagenesis. Constructs for the SBP2L SID and RBD were obtained by PCR amplifying coding regions corresponding to residues 467–647 and residues 648–1101 and TOPO-TA cloning the PCR product into pTrcHis. His-FLAG tagged SBP2L RBD was obtained by site-directed mutagenesis to convert the Xpress tag in pTrcHis to FLAG.

The predicted amino acid sequence of *Capitella* SBP2L was previously reported [17] and the sequence was refined by using ESTs EY604926.1, EY608589.1, and EY608588.1. Protein sequence conflicts between EY608589.1 and EY608588.1 were resolved by consulting *Capitella* genomic DNA sequence. After refinement the region encoding CT-SBP2L was synthesized with a Pac I site 3′ of the stop codon and optimization for mammalian codon usage and cloned into pUC57 by GeneWiz (South Plainfield, NJ). *Capitella* CT-SBP2L was then subcloned into the Kpn I/Pac I sites of a luciferase construct in pcDNA3.1. A full-length human SBP2 clone (BC036109) was obtained from ATCC and the coding region was PCR amplified and TOPO-TA cloned into pcDNA3.1. The coding region for human CT-SBP2 (a.a. 408–854) was PCR amplified from the full-length clone and TOPO-TA cloned into pcDNA3.1 or pTrcHis. SBP2 SID and RBD constructs were described previously [14]. Empty pcDNA3.1 was obtained by performing a mock TOPO-TA cloning reaction and PCR screening clones with vector primers. Wild-type and core deleted rat GPX4 SECIS elements have been described previously [7]. The AAA ⊙ TTT mutant was generated by site directed mutagenesis. Human SECIS elements were PCR amplified with Pac I/Not I linkers from oligo-dT primed cDNA from PC3 and RWPE cells, HeLa cell genomic DNA (a gift from the Dougherty lab), and synthetic oligonucleotide ultramers (Integrated DNA Technologies) and TOPO-TA cloned into pcDNA3.1. The luciferase reporter with a UGA codon at position 258 and rat GPX4 SECIS has been previously described [28]. The *Capitella* SelT SECIS flanked by Pac I and Not I sites was obtained by gene synthesis (Genewiz). The human GPX4 and SelV SECIS and *Capitella* SelT constructs were digested with Pac I and Not I and the SECIS fragment was cloned into Pac I/Not I digested luciferase reporter plasmid. Nonsilencing and SBP2L-targeting shRNAs in pGIPz were purchased from Open Biosystems. All constructs were confirmed by automated DNA sequencing.

### Recombinant proteins

All assays in this report using recombinant proteins utilized N-terminally 6xHis-Xpress tagged protein unless otherwise noted. All recombinant proteins were expressed and purified from in *E. coli* BL21 as described elsewhere [14], [29]. 6xHis-FLAG tagged SBP2L RBD was purified by nickel affinity chromatography as described for the 6xHis-Xpress tagged proteins. After purification proteins were dialyzed into 1× PBS+10% glycerol. Following dialysis proteins were centrifuged at full speed at 4°C to pellet insoluble material and then DTT was added to a final concentration of 2 mM.

### *In Vitro* transcription and translation

CT-SBP2L and CT-SBP2 expression plasmids (pCDNA3.1) were linearized with Xho I and used as templates to transcribe capped mRNAs with the T7 mMessage/mMachine kit (Ambion) according to the manufacturer's protocol. Luciferase reporter plasmids bearing the rat GPX4, human GPX4, human SelV, and *Capitella* SelT SECIS elements were linearized with Xho I or Not I. Messenger RNAs were extracted with phenol:chloroform:isoamyl alcohol (25∶24∶1), purified on P30 gel filtration spin columns (BioRad), and quantitated by UV spectrophotometry.

CT-SBP2 and CT-SBP2L *in vitro* transcribed mRNAs were translated at a final concentration of 20 ng/µL in nuclease treated rabbit reticulocyte lysate (Promega) in the presence of [^35^S]-Met according to the manufacturer's protocol. Mock reactions were supplemented with water instead of mRNA. Translation reactions were incubated at 30°C for 1 hour and 2 µL were fractionated by SDS-PAGE and visualized by phosphorimaging to confirm protein synthesis. In vitro translated proteins were quantitated by densitometry using ^35^S Met standards and assuming an endogenous Met concentration of 5 mM in rabbit reticulocyte lysate.

### Sec incorporation assay

Selenocysteine incorporation was monitored by translating 50 ng luciferase reporter mRNA in nuclease treated rabbit reticulocyte lysate supplemented with the indicated amounts of recombinant protein or 2 µL of [^35^S]-Met labeled *in vitro* translated protein. Final reaction volumes were 12.5 µL and were incubated for 1 hour at 30°C. [^35^S]-Met labeled reactions were analyzed for Sec incorporation by resolving 2 µL by SDS-PAGE and visualizing the translation products by phosphorimaging. The remainder of the reaction was stopped by addition of 50 µL 1× PBS and luciferase activity was measured with the firefly luciferase assay kit (Promega).

### SECIS probes and EMSA

SECIS containing plasmids were linearized with Hind III (rat GPX4 SECIS) or Not I (all human SECIS elements) and transcribed with T7 RNA polymerase in the presence of [^32^P]-α-UTP as described elsewhere [7]. Reactions (20 µL) containing 20 fmol SECIS RNA probe and the indicated amounts of recombinant protein in PBSDG (1× PBS, 10% glycerol, 2 mM DTT) were carried out in 1× PBS supplemented with 250 µg yeast tRNA (Sigma), 10 mM DTT, and 5 µg soybean trypsin inhibitor (Sigma) and incubated at 37°C for 30 min. Reactions containing the GPX6 SECIS element were also supplemented with 1 U/µL RNasin (Promega) because this SECIS was not stable in the absence of RNase inhibitor. After incubation, reactions were resolved on 4% nondenaturing polyacrylamide gels. The apparent dissociation constants were determined by generating a binding curve of the percentage of bound SECIS RNA and calculating the protein concentration at which 50% of the RNA was bound.

### UV cross-linking

Recombinant CT-SBP2 and CT-SBP2L were incubated with 20 fmol [^32^P]-α-UTP labeled SelV SECIS element as described for EMSAs. Following incubation, complexes were UV irradiated at 254 nm for 10 min and subsequently treated with 20 µg RNase A for 15 min at 37°C. Samples were resolved by 10% SDS-PAGE, and visualized by phosphorimaging.

### Domains in trans co-immunoprecipitation assay

40 pmol of SBP2 or SBP2L 6xHis-Xpress SID was incubated with equimolar amounts of FLAG-SBP2 RBD or 6xHis-FLAG SBP2L RBD and unlabeled *in vitro* transcribed SECIS elements as indicated in Figure 7 and Figure S2. SECIS elements were transcribed with the T7 Ribomax kit (Promega) using Hind III (rat GPX4) or Not I (human GPX4 and SelV) linearized plasmids as template. Complexes were formed in a final volume of 70 µL PBSDG and incubated at 30°C for 10 min. Reactions were then brought to a final volume of 200 µL with 1× PBS+1 mM DTT and 40 µL of 50% M2 α-FLAG agarose (Sigma) in the same buffer and rotated for 1 hour at 4°C. Complexes were pelleted at 2000× g for 1 minute at 4°C and 50% of the supernatant was saved for RNA extraction with TrizolLS (Invitrogen). A separate aliquot of supernatant was saved for western blot analysis. Pellets were washed 4×1 mL with 1× PBS+0.5% Tween-20. Portions equivalent to 50% of the pellet fraction and 7.5% of the supernatant were resolved by SDS-PAGE and analyzed by western blotting against SBP2 or SBP2L.

### SBP2L antibody

Polyclonal anti-SBP2L antibody was raised against recombinant 6xHis-Xpress CT-SBP2L in chickens (Pocono Rabbit Farm and Laboratory). Antibodies were purified from egg yolks by resuspending and stirring egg yolks with 10 volumes of water at 4°C and allowing the suspension to settle overnight. The soluble fraction was centrifuged for 10 min at 10,000× g and 4°C. Ammonium sulfate was added to the supernatant to a final concentration of 50% (291 g/L), mixed for 30 min at 4°C, and centrifuged for 10 min at 10,000× g and 4°C. Pellets were resuspended in 3–5 mL of 1× PBS and dialyzed against 3 changes of 1L 1× PBS at 4°C.

### Cell culture and transfection

All cell lines were maintained at 37°C with a humidified 5% CO2 atmosphere. HEK293 cells were cultured in DMEM+10% newborn calf serum. U87MG cells were cultured in MEM+10% FBS and PC3 cells in RPMI-1640+10% FBS. HEK293 cells stably transfected with pGIPz expressing shRNAs targeting SBP2L were selected with 2 µg/mL puromycin. Transient transfections were performed in HEK293 cells using FuGene6 (Roche) according to the manufacturer's protocol. Cells were seeded 24 hours prior to transfection at a density of 4×10^5^ per well in a 6 well plate and harvested 48 hours post-transfection.

### ^75^Se labeling

Eighteen to twenty-four hours prior to harvesting, cells were changed into medium containing ^75^Se (59.2 µCi/nmol) as 100 nM Na_2_SeO_3_. Cells were washed twice with cold PBS and lysed in RIPA buffer (20 mM Tris-HCl pH 8.0, 150 mM NaCl, 1% NP-40, 0.5 sodium deoxycholate, 0.1% SDS) containing 0.5 mM PMSF. Extracts were cleared by centrifugation at 17,000× g, 10 min, 4°C and protein concentration was determined by Bradford assay. Equal amounts of protein were fractionated by 15% SDS-PAGE and visualized by phosphorImaging or 10% SDS-PAGE for western blot analysis

### Immunoprecipitation and RT-PCR

U87MG cells: Twelve 100 mm dishes were cultured for 4 days, trypsinized, and washed two times in 10 mL cold 1× PBS. The cell pellet was resuspended in 2 mL IP buffer (20 mM Tris-HCl pH 7.5, 150 mM NaCl, 1.5 mM MgCl_2_, 1 mM EGTA, 1% Triton X-100, 0.5 mM PMSF) and rotated at 4°C for 10 min. Extracts were cleared by centrifugation at 17,000× g, 10 min, at 4°C. RNA was extracted from 100 µL of cleared extract with TrizolLS for input. The remaining extract was split evenly and supplemented with 40 µL of 50% anti-IgY agarose and pre-immune or α-SBP2L IgY. Complexes were immunoprecipitated overnight with rotation at 4°C then washed 3 times with 1 mL of IP buffer. On the final wash, 50% of each sample was transferred to a fresh tube. Proteins were eluted and submitted for MudPIT analysis by the Rutgers University mass spectrometry facility. The other set of samples was DNase treated prior to RNA extraction with TrizolLS. Extracted RNA (input and IP) was resuspended in 10 µL and quantitated with a NanoDrop 2000 spectrophotometer (ThermoScientific). PC3 cells: Two 100 mm dishes were supplemented with 50 nM Na_2_SeO_3_ and cultured for three days and harvested at ∼80% confluence. Cells were washed 2 times with 5 mL of ice-cold PBS and lysed by scraping in 1 mL of IP buffer followed by rotation for 10 min at 4°C. This extract was cleared by centrifugation at 17,000× g, 10 min, 4°C. Cleared extracts were aliquoted (200 µL/tube) and supplemented with 40 µL of a 50% slurry of protein A agarose or anti-IgY agarose and the appropriate antibody for SBP2 and SBP2L immunoprecipitation, respectively. Samples were processed as described for U87MG cells but mass spectrometric analysis was omitted. For RT-PCR, oligo-dT primed cDNAs were synthesized with SuperScriptIII reverse transcriptase (Invitrogen) according to the manufacturer's protocol using 100 ng input RNA or 10 ng immunoprecipitated RNA as template. One µL of cDNA was used as template for PCR amplification of the indicated genes for 30 cycles.

## Supporting Information

Figure S1 **Capitella CT-SBP2L.** Amino acid sequence of *Capitella* CT-SBP2L used in Figure 1. Black arrows correspond to ESTs. Asterisk represents the stop codon in the EST.
(EPS)

Figure S2 **The SelV SECIS does not promote stable SBP2L domain association.** (A) 6xHis-Xpress SBP2L SID was incubated with 6xHis-FLAG SBP2L was incubated with no SECIS (−), rat (r) or human (h) GPX4 SECIS, or SelV SECIS and complexes were immunoprecipitated (IP) with α-FLAG agarose. Pellet fractions were analyzed by western blot (WB) for SBP2L. (B) Same as in panel A but with 6xHis-Xpress SBP2 SID and FLAG SBP2 RBD. (C) Stained agarose gel of RNA extracted from the supernatants of A and B.
(EPS)

Figure S3 **Deleting the conserved inter-domain linker and C-terminal extension do not activate CT-SBP2L.** (A) SDS-PAGE gel of *in vitro* translated [^35^S]-Met labeled input proteins. (B) Luciferase activity obtained from *in vitro* translation of the diagrammed reporter mRNA in the presence of the input proteins from panel A.
(EPS)

Figure S4 **Validation of SBP2L antibody for immunoprecipitation.** SBP2L was immunoprecipitated from U87MG cell extracts and detected by western blot.
(EPS)
